# Estimating the Solubility, Solution Thermodynamics, and Molecular Interaction of Aliskiren Hemifumarate in Alkylimidazolium Based Ionic Liquids

**DOI:** 10.3390/molecules24152807

**Published:** 2019-08-01

**Authors:** Md. Khalid Anwer, Mohammad Muqtader, Muzaffar Iqbal, Raisuddin Ali, Bjad K. Almutairy, Abdullah Alshetaili, Saad M. Alshahrani, Mohammed F. Aldawsari, Ahmed Alalaiwe, Faiyaz Shakeel

**Affiliations:** 1 Department of Pharmaceutics, College of Pharmacy Prince Sattam Bin Abdulaziz University, Al-Alkharj, 11942, Saudi Arabia; 2 Department of Pharmaceutical Chemistry, College of Pharmacy, King Saud University, Riyadh 11451, Saudi Arabia; 3 Bioavailability Laboratory, College of Pharmacy, King Saud University, Riyadh 11451, Saudi Arabia; 4 Department of Pharmaceutics and Central Lab, College of Pharmacy, King Saud University, Riyadh 11451, Saudi Arabia; 5 Department of Pharmaceutics, College of Pharmacy, King Saud University, Riyadh 11451, Saudi Arabia

**Keywords:** ionic liquids, solubility, thermodynamics, molecular interaction, entropy

## Abstract

Estimating the solubility and solution thermodynamics parameters of aliskiren hemifumarate (AHF) in three different room temperature ionic liquids (RTILs), Transcutol-HP (THP) and water are interesting as there is no solubility data available in the literature. In the current study, the solubility and solution thermodynamics of AHF in three different RTILs, THP and water at the temperature range from 298.2 to 318.2 K under air pressure 0.1 MP were evaluated. The solid phase evaluation by Differential Scanning Calorimetry (DSC) and Powder X-ray Diffraction (PXRD) indicated no conversion of AHF into polymorph. The mole fraction solubility of AHF was found to be highest in 1-hexyl-3-methylimidazolium hexafluorophosphate (HMMHFP) ionic liquid (7.46 × 10^−2^) at 318.2 K. The obtained solubility values of AHF was regressed by the Apelblat and van’t Hoff models with overall root mean square deviations (*RMSD*) of 0.62% and 1.42%, respectively. The ideal solubility of AHF was higher compared to experimental solubility values at different temperatures. The lowest activity coefficient was found in HMMHFP, which confirmed highest molecular interaction between AHF–HMMHFP. The estimated thermodynamic parameters confirmed endothermic and entropy driven dissolution of AHF in different RTILs, THP, and water.

## 1. Introduction

**Figure 1:**
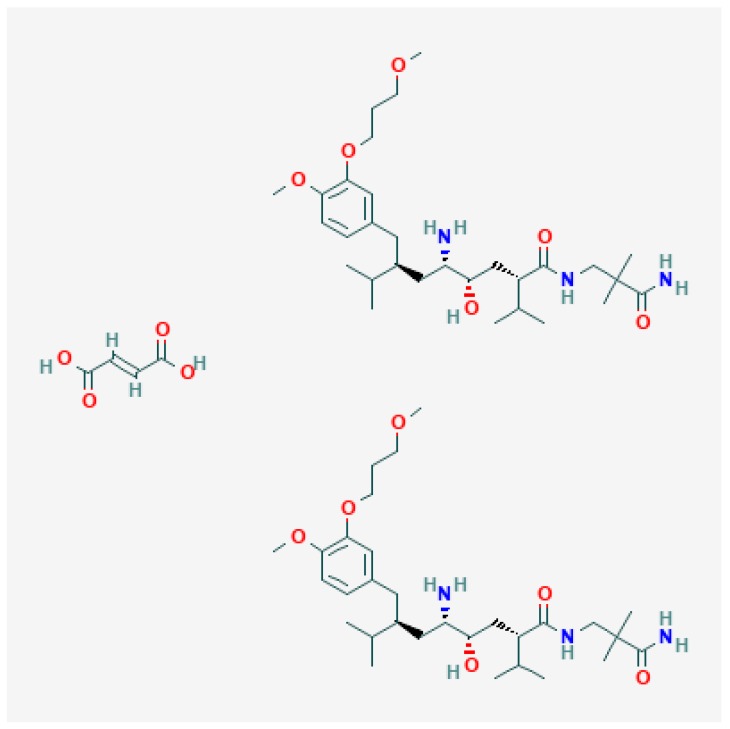
Molecular structure of aliskiren hemifumarate (AHF) (molar mass: 1219.61 g mol^−1^).

Renin is an enzyme secreted by the kidney and plays a key role in the pathogenesis of arterial hypertension and its consequent complication associated with cardiovascular and renal diseases [1]. Aliskiren hemifumarate (AHF) is the first clinically approved, orally active, highly potent, and selective renin inhibitor for the treatment of hypertension alone or in combination with other antihypertensive agents [2]. It is chemically designated as (2*S*,4*S*,5*S*,7*S*)-*N*-(2-Carbamoyl-2-methylpropyl)-5amino-4-hydroxy-2,7-diisopropyl-8-[4-methoxy-3-(3-methoxypropoxy)phenyl]-octanamidehemifumarate (Figure 1). It is white to slightly yellowish crystalline powder having a molecular weight of 1219.61. In spite of high potency, aliskiren is poorly absorbed (2.5% oral bioavailability) and reflects a high inter-subject variability in their pharmacokinetic profile after oral administration [3,4]. Due to poor bioavailability, a comparatively higher dose of aliskiren (150–300 mg) is required for therapeutic management of hypertension. In addition, high fat food decreases the absorption of aliskiren and therefore patients use to establish a routine pattern for taking aliskiren with regard to meals (US PI).

**Figure 2:**
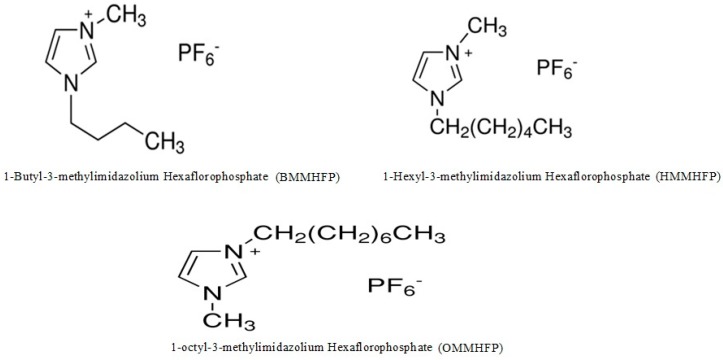
Chemical structure of alkyl imidazolium-based room temperature ionic liquids (RTILs).

Room temperature ionic liquids (RTILs) are basically organic molten salts which exist in the liquid state at room temperatures [5]. RTILs are usually prepared by combining large organic cations with a variety of smaller organic or inorganic anions. It has many unique physicochemical properties in comparison to traditional solvents and therefore emerged as a multifunctional solvent for pharmaceutical and biomedical applications [6,7,8,9]. It exhibits very low/or negligible vapor pressure, low melting point with a wide liquidity range, a high thermal, chemical & electrochemical stabilities, unique solvating properties with excellent dissolution performance [10,11,12]. In addition, it has adjustable solvent viscosity, polarity, and hydrophobicity associated with low or high water miscibility which results from a matchless association of molecular characteristics of their constitutive ions [7,8]. Moreover, some RTILs behave as nano segregated fluids with polar networks permeated by a polar domain, which enables the understanding of their peculiar solvent behavior at a molecular level [9]. RTILs have been utilized for solubility enhancement of poorly aqueous soluble drug and efficacy enhancer of topical analgesic with increased thermal stability properties [13,14]. Therefore, RTILs can overcome various pharmaceutical problems, i.e., low solubility, low permeability, which results in low bioavailability of drugs, and the presence of polymorph/solvate, which severely effects the efficacy of commercially available drugs [8]. Among the variety of RTILs, alkylimidazolium-based RTILs, in which the alkyl group of the ILs can be varied by selection of different halohydrocarbons is most commonly employed RTILs, and have become one of the research hot topics in recent years [6]. The solubility data of AHF in safe/non-toxic ionic liquids measured in this study could be useful in pharmaceutical dosage form development. In this study, we used three different alkyl imidazolium-based RTILs (Figure 2). Therefore, the aim of this study was to evaluate the solubility, solution thermodynamic, and molecular interaction of AHF with different alkyl imidazolium-based RTILs.

## 2. Results and Discussion

### 2.1. Solid Phase Characterization of AHF

**Figure 3:**
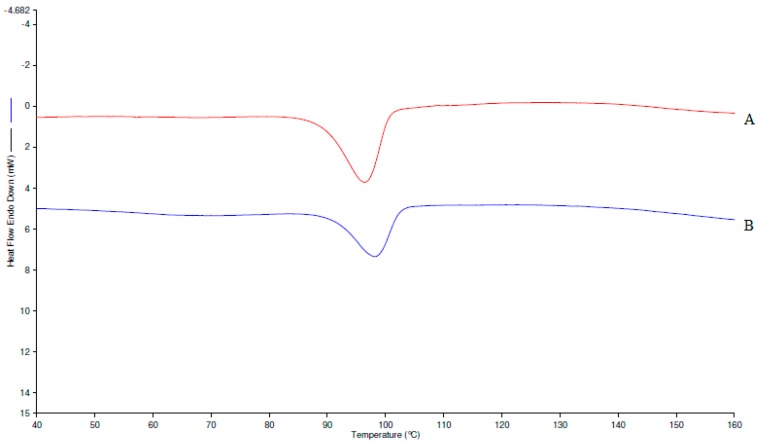
Differential Scanning Calorimetry (DSC) spectra of (**A**) pure AHF and (**B**) equation equilibrated AHF (recovered from water).

**Figure 4:**
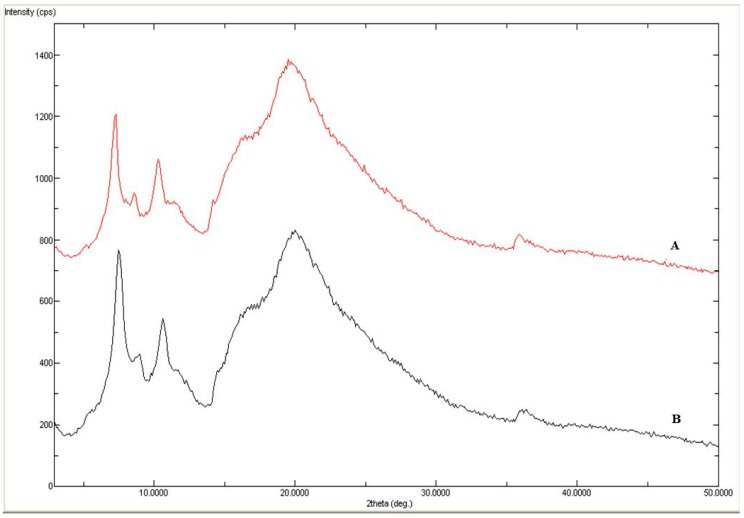
Powder X-ray Diffraction (PXRD) spectra of (**A**) pure AHF and (**B**) equation equilibrated AHF (recovered from water).

Solid phase characterization of the initial and equilibrated sample were performed by Differential Scanning Calorimetry (DSC) and Powder X-ray Diffraction (PXRD) spectral analysis to know about the crystallinity/amorphicity and various thermal parameters of the drug. The DSC spectra of initial and equilibrated AHF (obtained from water) are shown in Figure 3A,B, respectively. The DSC spectra of initial AHF showed a broad peak at the fusion temperature (*T*_fus_) of 369.62 K, confirming AHF’s amorphous nature (Figure 3A). However, an equilibrated sample of AHF showed almost similar peaks at 371.62 K which suggested its amorphous nature (Figure 3B). The enthalpy fusion (∆*H*_fus_) of initial and equilibrated AHF were measured as 36.85 kJ mol^−1^ and 38.21 kJ mol^−1^, respectively. The DSC thermograms of initial and equilibrated AHF were found to be almost similar and did not convert into polymorphs after reaching equilibrium. The *T*_fus_ and ∆*H*_fus_ of AHF by DSC analysis has been available in literature as 371.5 K and 30.28 kJ mol^−1^, respectively [15], which were very close to our obtained data. The studied temperature range of 298.2 to 318.2 K was selected in the present work in such a way that the maximum studied temperature (i.e., *T* = 318.2 K) should not exceed the melting/fusion temperature of AHF. The melting temperature of AHF was obtained as 371.5 K in this work. The maximum studied temperature was much lower than the melting temperature of AHF, hence, the proposed temperature range was selected in this study. The XRD diffraction pattern of initial and equilibrated AHF are presented in Figure 4A and B, respectively. The diffraction pattern of initial AHF showed broad and diffused peaks suggesting its amorphous nature (Figure 4A), as it has been also confirmed by DSC studies. However, the same broad and diffused peaks could be seen in the equilibrated sample, suggesting no change after equilibrium [15].

### 2.2. Measured Solubilities of AHF

The obtained *x*_e_ data of AHF in RTILs, Transcutol-HP (THP) and water at *T* = 298.2 K, 303.2 K, 308.2 K, 313.2 K, and 318.2 K under atmospheric pressure are shown in Table 1. There is no literature available on solubility of AHF in the RTILs, which would be the replacement of hazardous organic solvents for the development of dosage form.

The solubility of AFH in water at T = 298.2 K is approximately 100 mg/mL (converted to 1.47 × 10^−3^ in mole fraction solubility) as reported in the literature [16]. While, solubility of AHF in RTILs and THP with respect to temperature is not reported elsewhere. The mole fraction solubility of AHF in water at *T* = 298.2 K was found to be 1.45 × 10^−3^ which was much closer to reported solubility [16]. It was noted in our studies that mole fraction solubilities (*x*_e_) of AHF increased with increases in temperature in experimental solvents. The *x*_e_ values of AHF were obtained highest in HMMHFP followed by BMMHFP, THP, OMMHFP, and water at *T* = 298.2 K. The same trends were also seen at all studied temperatures (*T* = 303.2–318.2 K) (see Table 1). The highest solubility of AHF was observed in RTILs probably due to anion–cation combinations of RTILs, which are responsible for H-bonding, Van der Waals forces, and π–π interactions between solute and solvent [17]. Based on solubility studies, it is considered that AHF is freely soluble in all investigated solvents, as AHF is an amorphous powder, confirmed by DSC and PXRD.

### 2.3. Hansen Solubility Parameters

Hansen solubility parameters (*δ*) are very useful to correlate the solubility data of AHF with polarity of AHF and solvents. The values of *δ* for AHF and RTILs, THP and water were calculated with the help of Equation (1) [17]:

(1) $$\delta^{2}=\delta_{d}^{2}+\;\delta_{p}^{2}+\;\delta_{h}^{2}$$

In this Equation, *δ*_d_, *δ*_p,_ and *δ*_h_ represent dispersion, polar, and hydrogen-bonded parameters for Hansen solubility, respectively. The HSPiP software (version 4.1.07) was used to estimate these parameters (*δ*, *δ*_d_, *δ*_p,_ and *δ*_h_). The estimated data of these parameters are given in Table 2.

The *δ* value for AHF was obtained as 25.00 MPa^1/2^, suggesting that it is drug with medium polarity. The *x*_e_ values of AHF were also found to be higher in all RTILs and THP as these RTILs and THP have medium *δ* values (Table 2). The *x*_e_ value of AHF was recorded as a maximum in HMMHFP. This observation was probably due to the close *δ* value of HMMHFP (22.70 MPa^1/2^) with that of AHF. However, the *x*_e_ value of AHF was found minimum in water and this observation was probably due to the maximum *δ* value of water (47.80 MPa^1/2^).

### 2.4. Measurement of Ideal Solubilities and Activity Coefficients

The ideal solubility of pure AHF (*x*^idl^) was measured by using equation (2) [18]:

(2) $$\ln\;x^{\mathrm{idl}}=\;\frac{-\Delta H_{\mathrm{fus}}\left(T_{\mathrm{fus}}-T\right)}{RT_{\mathrm{fus}}T}+\left(\frac{\Delta C_{p}}{R}\right)[\frac{T_{\mathrm{fus}}-T}{T}+\ln\left(\frac{T}{T_{\mathrm{fus}}}\right)].$$

In this equation, *R* = universal gas constant and ∆*C*_p_ = difference in the molar heat capacity of solid form with that of liquid form [18,19]. The ∆*C*_p_ value for AHF was approximately calculated using Equation (3) [18]:

(3) $$\Delta C_{p}=\;\frac{\Delta H_{\mathrm{fus}}}{T_{\mathrm{fus}}}$$

The values of *T*_fus_ and ∆*H*_fus_ for pure powder AHF were measured by DSC thermal analysis and recorded as 369.62 K and 36.85 kJ mol^−1^, respectively. By applying Equation (4), the ∆*C*_p_ data for AHF was measured as 99.69 J mol^−1^ K^−1^. The measured data of *x*^idl^ are documented in Table 2. The ideal solubilities of AHF were measured much higher in comparison to mole fraction solubilities of AHF in different RTILs, THP, and water at all investigated temperatures. The ideal solubilities of AHF were measured in the range of 7.61 × 10^−2^ to 1.65 × 10^−1^. The higher ideal solubilities of AHF could be due to lower fusion temperature of amorphous AHF powder. Among the different solvents studied, RTILs (HMMHFP) was found to be the ideal solvent for the solubilization of AHF.

The activity coefficients data of (*γ*_i_) for AHF in all experimental solvents were measured by using Equation (4) [19,20]:

(4) $$\gamma_{i}=\;\frac{x^{\mathrm{idl}}}{x_{e}}$$

The values of activity coefficients (*γ*_i_) of AHF were investigated in all solvents at *T* = 298.2 K, 303.2 K, 308.2 K, 313.2 K, and 318.2 K and are documented in Table 3. The values of the activity coefficients (*γ*_i_) of AHF are very important parameters to explain solute–solvent interaction at the molecular level. The highest value of *γ*_i_ for AHF in water was determined in comparison to other investigated solvents. While, data of *γ*_i_ for AHF was found much lower in RTILs (HMMHFP) as it could be seen in Table 3. A slight fluctuation in *γ*_i_ was observed in water with change in temperatures. While, *γ*_i_ values were found almost constant in other investigated solvents with a variation in temperatures. The determined *γ*_i_ data for AHF were measured in accordance with mole fraction solubility in all solvents studied. According to *γ*_i_ values, the highest molecular interactions were obtained in AHF–HMMHFP in comparison to other solute–solvent interactions. While, the weak molecular interactions were obtained in AHF–water due to its highest *γ*_i_ obtained values in water.

### 2.5. Correlation of x_e_ Values of AHF

The obtained *x_e_* values of AHF in this work were correlated by using the Apelblat and van’t Hoff models [21,22,23,24]. Apelblat model solubility (*x*^Apl^) for AHF was calculated by using Equation (5) [20,23]:

(5) $$\ln x^{\mathrm{Apl}}=A+\frac{B}{T}+\;C\ln\left(T\right)$$

In this Equation, the symbols *A*, *B* and *C* are parameters of the Apelblat model which were calculated by non-linear multivariate regression analysis of *x_e_* values of AHF and are listed in Table 4. The values *x_e_* of AHF were correlated with *x*^Apl^ of AHF in terms of the root mean square deviations (*RMSD*) and *R^2^.* The values of *RMSD* were calculated by using Equation (6):

(6) $$\ln x^{\mathrm{Apl}}=A+\frac{B}{T}+\;C\ln\left(T\right)\;RMSD=\;{\left[\frac{1}{N}\overset{N}{\underset{i=1}{\sum }}{\left(\frac{x^{\mathrm{Apl}}-x_{e}}{x_{e}}\right)}^{2}\right]}^{\frac{1}{2}}$$

**Figure 5:**
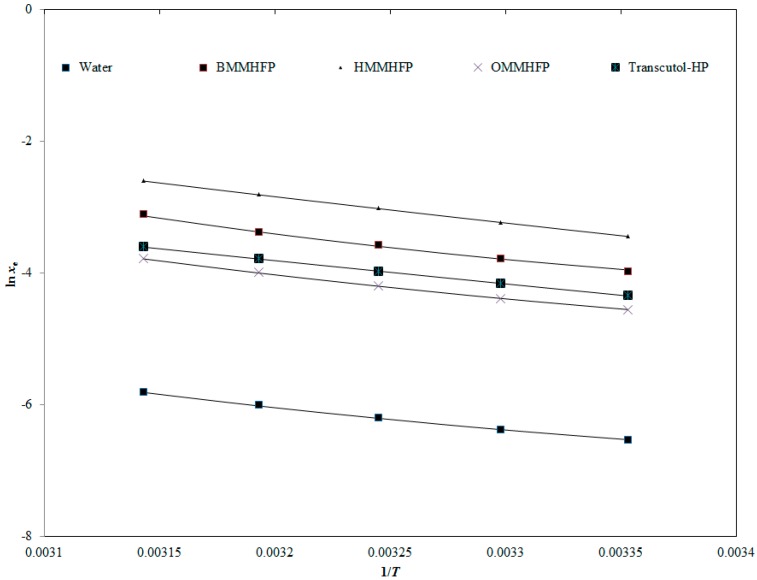
Correlation of experimental natural logarithmic solubilities (ln*x*_e_) of AHF with the Apelblat model in three different ILs, water, and Transcutol-HP as a function of 1/*T*; symbols represent the experimental ln*x*_e_ values of AHF and the solid lines represent the ln*x*^Apl^ values calculated by the Apelblat model.

In this equation, N is the number of experimental data points. The correlation between values of In*x_e_* and In*x*^Apl^ of AHF against 1/T are given in Figure 5. It could be seen in Figure 5 that a good correlation/curve fitting obtained between values of In*x_e_* and In*x*^Apl^ of AHF in all investigated solvents. The measured values of “*A*, *B*, *C*, *RMSD*, and *R^2^*” are listed in Table 5. The obtained values of *RMSD* in different solvents were found in the range of 0.57% to 0.91%. The overall *RMSD* for Apelblat correlation was measured as 0.62%. The highest (0.91%) and lowest (0.57%) *RMSD* values for AHF were obtained in BMMHFP and OMMHFP, respectively. The obtained *R^2^* values for AHF were found as 0.9996 to 0.9999. These obtained data revealed an excellent curve fitting of values of *x_e_* and *x*^Apl^ for AHF using the Apelblat model.

The van’t Hoff model solubility (*x*^van’t^) for AHF were calculated using Equation (7) [22]:

(7) $$\ln x^{{\mathrm{van}}^{\prime }t}=a+\frac{b}{T}.$$

In the above Equation, symbols *a and b* are coefficients of the van’t Hoff model.

**Figure 6:**
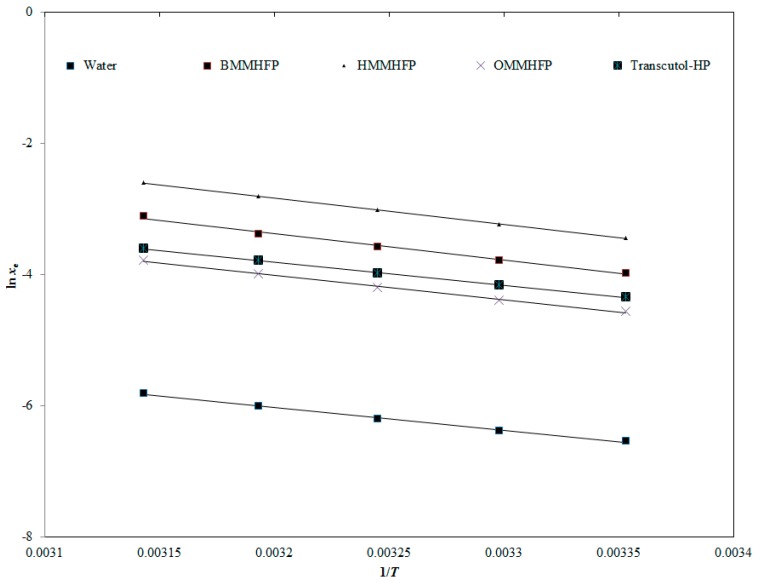
Correlation of experimental natural logarithmic solubilities (ln*x*_e_) of AHF with the van’t Hoff model in three different ILs, water, and Transcutol-HP as a function of 1/*T*; symbols represent the experimental ln*x*_e_ values of AHF and the solid lines represent the ln*x*^van’t^ values calculated by the van’t Hoff model.

These coefficients values were obtained by simple regression of graphs plotted for In*x_e_* data of AHF against 1/T. The measured *x_e_* values of AHF also correlated with *x*^van’t^ values using *RMSD* and *R^2^* values. The curve correlation between *x_e_* and *x*^van’t^ values of AHF as a function of 1/T is given in Figure 6. The data represented in Figure 6 again showed an excellent correlation/curve fitting between *x_e_* and *x*^van’t^ values of AHF. The calculated values of a, b, *RMSD*, and *R^2^* are documented in Table 5. The *RMSD* values for AHF in all investigated solvents were obtained as 0.74% to 1.70%. The overall *RMSD* was obtained as 1.42%. The maximum and minimum values of RMSD for AHF were obtained in the RTILs (HMMHFP) as 1.70% and RTILs (OMMHFP) 0.70%, respectively. The coefficient of correlation (*R^2^*) values for AHF in all investigated solvents were calculated as 0.9953 to 0.9998. These results again indicated an excellent curve fitting of *x_e_* values of AHF with the van’t Hoff model.

### 2.6. Measurement of Apparent Thermodynamic Parameters

The solubilization behavior of AHF in all investigated solvents was studied by determination of apparent standard thermodynamic parameters. The thermodynamic parameters namely: apparent standard dissolution enthalpy (Δ_sol_*H*^0^), apparent standard Gibbs free energy (Δ*_sol_G^0^*) and apparent standard dissolution entropy (Δ*_sol_S^0^*) for dissolution behavior were obtained with the help of apparent thermodynamic analysis. The Δ*_sol_H^0^* data for AHF solubilization in all solvents were determined at the “mean harmonic temperature (*T_hm_*) with the help of van’t Hoff analysis using Equation (8) [20,25].

(8) (∂ln xe∂(1T−1Thm))P= −ΔsolH0R

The “Δ*_sol_H^0^*” values for dissolution of AHF in all solvents were calculated by van’t Hoff curve constructed for In*x_e_* values of AHF as a function of *1/T − 1/T*_hm._ The Δ*_sol_G^0^* values for dissolution behavior of AHF in all investigated solvents were estimated at *T*_hm_ by using Krug et al. analysis using Equation (9) [26]:

(9) $$\Delta_{\mathrm{sol}}G^{0}=-RT_{\mathrm{hm}}\times intercept$$

The value of intercept for AHF in each investigated solvent was calculated from thevan’t Hoff plot. The values of Δ*_sol_S^0^* for dissolution behavior of AHF in all investigated solvents were calculated with the help of combined approach of van’t Hoff and Krug et al. analysis using Equation (10) [26,27].

(10) $$\Delta_{\mathrm{sol}}S^{0}=\;\frac{\Delta_{\mathrm{sol}}H^{0}-\Delta_{\mathrm{sol}}G^{0}}{T_{\mathrm{hm}}}$$

The obtained values of Δ_sol_*H^0^*, Δ_sol_*G^0^*, Δ_sol_*S^0^*, and *R^2^* for dissolution behavior of AHF in all solvents are documented in Table 6. The Δ_sol_*H^0^* values for dissolution behavior of AHF in all investigated solvents were calculated as 29.01–33.41 kJ/mol. The highest and lowest Δ_sol_*H^0^* values for AHF were obtained as 33.41 kJmol^−1^and 29.01 kJ/mol in BMMHFP and water, respectively. The Δ_sol_G^0^ values for AHF solubilization in different investigated solvents were observed as 7.71–15.83 kJ/mol. The Δ_sol_G^0^ values for AHF solubilization was observed highest in water followed by OMMHFP, THP, BMMHFP, and HMMHFP solvents. The lowest values of Δ_sol_G^0^ for AHF solubilization in HMMHFP ionic liquids, which was probably due to the highest solubility of AHF in HMMHFP. The positive obtained values of Δ_sol_*H^0^* and Δ_sol_*G^0^* for AHF solubilization in all investigated solvents confirmed an endothermic dissolution of AHF [22,24]. The Δ_sol_*S^0^* values for AHF solubilization behavior in all solvents was obtained in positive values as 42.80–83.15 kJ/mol. The average values of Δ_sol_*H^0^*, Δ_sol_*G^0^*, and Δ_sol_*S^0^* were calculated as 31.22 kJ/mol, 10.70 kJ/mol, and 66.62 kJ/mol with relative uncertainties of 0.06 kJ/mol, 0.28 kJ/mol, and 0.23 kJ/mol, respectively. The positive Δ_sol_*S^0^* values for AHF in all solvents indicated an entropy driven solubilization [22,27].

## 3. Materials and Methods

### 3.1. Materials

AHF, BMMHFP, HMMHFP, and OMMHFP were purchased from Mesochem Technology (Beijing, China). Transcutol-HP^®^ was obtained from Gattefosse (Lyon, France). Purified water (conductivity less than 1 µS·cm^−1^) was taken from Milli-Q Water Purification Unit in College of Pharmacy, Prince Sattam Bin Abdul-Aziz University, Al-Kharj Saudi Arabia. The data of physical properties and characterization used in the experiment are listed in Table 7.

### 3.2. Analysis of AHF

The samples were quantified by using UV Spectroscopy (JASCO, Japan) at lambda max of 280 nm after dilution with suitable solvents [26]. A linear standard curve of AHF was plotted between concentration 10–150 µg g^−1^ and absorbance with coefficient of correlation of *R*^2^ = 0.9990. A regression equation was obtained as y = 0.005x + 0.010 (where, y = measured absorbance of AHF and x = concentration of AHF). The concentrations of AHF samples were determined with help of regression equation of standard plot.

### 3.3. Solid Phase Characterization of AHF

Solid phase characterization of initial and equilibrated AHF was performed by Differential Scanning Calorimetry (DSC) and Powder X-ray Diffraction (PXRD) analysis. DSC and PXRD evaluation were done to know the many thermal properties of initial and equilibrated samples of AHF [26,27,28]. DSC spectral analysis of the samples were recorded by Perkin Elmer DSC-8000, Waltham, MA, USA equipped with autosampler and controlled by Pyris software (Waltham, MA, USA). Accurately weighed amount of initial AHF (5mg) and equilibrated AHF (5mg) was pressed into hermetically sealed aluminum pan. DSC spectral analysis of both samples were performed in inert nitrogen gas environment with a flow rate of 50 mL/min. The temperature scan was used as 10 degree min^−1^ *T* = 313.2–433.2 K. PXRD spectral analysis of both AHF sample were performed on instrument Ultima IV (Rigaku Inc., Tokyo, Japan). The 2θ values were set in the range of 3° to 120° with a scan rate of 0.5 degree/min. The powder XRD of both samples were analyzed at a “tube voltage of 40 kV and 40 mV current”.

### 3.4. Solubility Studies of AHF

Mole fraction solubility of AHF in three different room temperature ionic liquids (BMMHFP, HMMHFP, and OMMHFP), transcutol-HP, and water was determined by shake flask method [29,30]. The solubility of AHF in these solvents was measured at T = 298.2 K, 303.2 K, 308.2 K, 313.2 K, and 318.2 K under atmospheric pressure. In brief, excess amount of AHF drug was dispersed in each investigated solvents enclosed in screw capped glass flask. Each AHF solution mixture was kept on a biological shaker (Julabo, Allentown, PA, USA) for shaking at 100 rpm for 72 h to reach equilibrium. After complete saturation of AHF in each solvents, sample was taken from shaker and kept to sediment the drug particles [17]. Then, required volume of supernatant samples were withdrawn, filtered and analyzed the AHF after suitable dilution with respective solvents by UV spectrophotometry at 280 nm [26]. Each experiments were done in triplicate.

Mole fraction solubilities of AHF (*x*_e_) were calculated with the help of Equation (11) [30].

(11) $$x_{e}=\;\frac{m_{1}/M_{1}}{m_{1}/M_{1}+m_{2}/M_{2}}$$

Here, *m*_1_ and *m*_2_ represents the masses of AHF and solvents used, respectively. *M*_1_ and *M*_2_represent the molar masses of AHF and solvents used, respectively.

## 4. Conclusions

In this study, the solubility of AHF in different alkyl imidazolium-based ionic liquids, THP and water were measured at 298.2–318.2 K. The solubility was found to increase with respect to increases in temperature in all investigated solvents. The obtained solubility data were well fitted/correlated with the Apelblat and van’t Hoff models. The data confirmed that solubilization of aliskiren hemifumarate was endothermic, spontaneous, and entropy driven in all solvents. In conclusion, aliskiren hemifumarate has been considered free soluble in all solvents due to its amorphous nature as confirmed by DSC/PXRD. However, maximum solubility was observed in 1-hexyl-3-methylimidazolium hexafluorophosphate (HMMHFP) ionic liquid.

## Figures and Tables

**Table 1:** Experimental solubilities (*x*_e_) of AHF in three different Ionic Liquids (ILs), Transcutol-HP, and water at *T* = 298.2–318.2 K and *p* = 0.1 MPa ^a^.

Sample	*x*_e_				
	*T* = 298.2 K	*T* = 303.2 K	*T* = 308.2 K	*T* = 313.2 K	*T* = 318.2 K
BMMHFP	1.89 × 10^−2^	2.29 × 10^−2^	2.80 × 10^−2^	3.42 × 10^−2^	4.47 × 10^−2^
HMMHFP	3.22 × 10^−2^	3.95 × 10^−2^	4.91 × 10^−2^	6.08 × 10^−2^	7.46 × 10^−2^
OMMHFP	1.04 × 10^−2^	1.24 × 10^−2^	1.51 × 10^−2^	1.86 × 10^−2^	2.29 × 10^−2^
Transcutol-HP	1.30 × 10^−2^	1.57 × 10^−2^	1.89 × 10^−2^	2.28 × 10^−2^	2.72 × 10^−2^
Water	1.45 × 10^−3^	1.69 × 10^−3^	2.03 × 10^−3^	2.47 × 10^−3^	3.01 × 10^−3^
*x* ^idl^	7.61 × 10^−2^	9.29 × 10^−2^	1.13 × 10^−1^	1.37 × 10^−1^	1.65 × 10^−1^

^a^ The standard uncertainties *u* are *u*(*T*) = 0.25 K, *u*(*p*) = 0.003 MPa, and *u*_r_(*x*_e_) = 1.47%.

**Table 2:** The *δ* value of AHF, three different ILs, Transcutol-HP and water at *T* = 298.2 K calculated by HSPiP software.

Sample	Hansen Solubility Parameters			
	*δ*_d_ (MPa^1/2^)	*δ*_p_ (MPa^1/2^)	*δ*_h_ (MPa^1/2^)	*δ* (MPa^1/2^)
AHF	19.20	10.80	11.90	25.00
BMMHFP	16.40	3.90	10.40	19.80
HMMHFP	16.30	10.60	11.60	22.70
OMMHFP	15.50	3.30	8.10	17.80
Transcutol-HP	16.30	7.20	11.90	21.40
Water	15.50	16.00	42.30	47.80

**Table 3:** The values of *γ*_i_ for AHF in three different ILs, Transcutol-HP, and water at *T* = 298.2 K to 318.2 K calculated using *x*^idl^ and *x*_e_ values.

Sample	*γ*_i_				
	*T* = 298.2 K	*T* = 303.2 K	*T* = 308.2 K	*T* = 313.2 K	*T* = 318.2 K
BMMHFP	4.02	4.04	4.02	4.01	3.70
HMMHFP	2.37	2.35	2.30	2.26	2.22
OMMHFP	7.26	7.49	7.48	7.36	7.24
Transcutol-HP	5.84	5.92	5.98	5.99	6.08
Water	52.36	54.83	55.61	55.43	54.95

**Table 4:** Apelblat parameters (*A*, *B*, and *C*), *R*^2^, and *RMSD* (%) for AHF in three different ILs, Transcutol-HP, and water.

Sample	*A*	*B*	*C*	*R* ^2^	*RMSD* (%)	Overall *RMSD* (%)
BMMHFP	−820.40	34062.78	123.24	0.9990	0.91	
HMMHFP	−147.87	3229.04	23.44	0.9999	0.74	
OMMHFP	−546.91	21723.32	82.40	0.9996	0.57	0.62
Transcutol-HP	−77.57	370.85	12.63	0.9998	0.76	
Water	−596.20	24103.49	89.30	0.9996	0.87	

**Table 5:** van’t Hoff model parameters (*a* and *b*), *R*^2^, and *RMSD* (%) for AHF in three different ILs, Transcutol-HP, and water.

Sample	*a*	*B*	*R* ^2^	*RMSD* (%)	Overall *RMSD* (%)
BMMHFP	9.47	−4013.50	0.9929	1.54	
HMMHFP	9.98	−4003.70	0.9997	1.70	
OMMHFP	7.93	−3730.70	0.9964	0.74	1.42
Transcutol-HP	7.46	−3521.40	0.9998	1.44	
Water	5.13	−3485.20	0.9953	1.69	

**Table 6:** Thermodynamic quantities (Δ_sol_*H*^0^, Δ_sol_*G*^0^, and Δ_sol_*S*^0^) and *R*^2^ values for AHF dissolution in three different ILs, Transcutol-HP, and water^b^.

Sample	Δ_sol_*H*^0^ (kJ mol^−1^)	Δ_sol_*G*^0^ (kJ mol^−1^)	Δ_sol_*S*^0^ (J mol^−1^ K^−1^)	*R* ^2^
BMMHFP	33.41	9.11	78.90	0.9931
HMMHFP	33.33	7.71	83.15	0.9997
OMMHFP	31.06	10.70	66.09	0.9965
Transcutol-HP	29.31	10.16	62.18	0.9998
Water	29.01	15.83	42.80	0.9954

^b^ The relative uncertainties are *u*(Δ_sol_*H*^0^) = 0.06 kJ mol^−1^, *u*(Δ_sol_*G*^0^) = 0.28 kJ mol^−1^, and *u*(Δ_sol_*S*^0^) = 0.23 J mol^−1^ K^−1^.

**Table 7:** Information about materials used in the experiment.

Material	Molecular Formula	Molar Mass (g mol^−1^)	CAS Registry No.	Purification Method	Mass Fraction Purity	Analysis Method	Source
AHF	C_64_H_110_N_6_O_16_	1219.61	173334-58-2	None	>0.99	HPLC	Synthesized
BMMHFP	C_8_H_15_F_6_N_2_P	284.18	174501-64-5	None	>0.99	HPLC	Sigma Aldrich
HMMHFP	C_10_H_19_F_6_N_2_P	312.24	304680-35-1	None	>0.99	HPLC	Sigma Aldrich
OMMHFP	C_12_H_23_F_6_N_2_P	340.29	304680-36-2	None	>0.99	HPLC	Fluka Chemica
Transcutol-HP	C_6_H_14_O_3_	134.17	111-90-0	None	>0.99	GC	Gattefosse
Water	H_2_O	18.07	7732-18-5	None	-	-	Milli-Q

Both the analysis method and purity were provided by supplier except for water.

## References

[1] Pantzaris N.D., Karanikolas E., Tsiotsios K., Velissaris D. (2017). Renin Inhibition with Aliskiren: A Decade of Clinical Experience. J. Clin. Med..

[2] Wood J.M., Maibaum J., Rahuel J., Grütter M.G., Cohen N.C., Rasetti V., Rüger H., Göschke R., Stutz S., Fuhrer W. (2003). Structure-based design of aliskiren, a novel orally effective renin inhibitor. Biochem. Biophys. Res. Commun..

[3] Waldmeier F., Glaenzel U., Wirz B., Oberer L., Schmid D., Seiberling M., Valencia J., Riviere G.J., End P., Vaidyanathan S. (2007). Absorption, Distribution, Metabolism, and Elimination of the Direct Renin Inhibitor Aliskiren in Healthy Volunteers. Drug. Metab. Dispos..

[4] Limoges D., Dieterich H.A., Yeh C.M., Vaidyanathan S., Howard D., Dole W.P. (2008). A study of dose-proportionality in the pharmacokinetics of the oral direct renin inhibitor aliskiren in healthy subjects. Int. J. Clin. Pharmacol. Ther..

[5] Halett P., Welton T. (2011). Room temperature ionic liquids: Solvents for synthesisand catalysis. Chem. Rev..

[6] Cao Y., Zhang R., Cheng T., Guo J., Xian M., Liu H. (2017). Imidazolium-based ionic liquids for cellulose pretreatment: Recent progresses and future perspectives. Appl. Microbiol. Biotechnol..

[7] Plechkova N.V., Seddon K.R. (2008). Applications of ionic liquids in the chemicalindustry. Chem. Soc. Rev..

[8] Marrucho I.M., Branco L.C., Rebelo L.P. (2014). Ionic liquids in pharmaceutical applications. Annu. Rev. ChemBiomol. Eng..

[9] Lopes J., Padua A.A.H. (2006). Nanostructural organization in ionic liquids. J. Phys. Chem. B.

[10] Berthod A., Ruiz-Ángel M.J., Carda-Broch S. (2018). Recent advances on ionic liquid uses in separation techniques. J. Chromatogr A.

[11] Fredlake C.P., Crosthwaite J.M., Hert D.G., Aki S.N.V.K., Brennecke J.F. (2004). Thermophysical Properties of Imidazolium-Based Ionic Liquids. J. Chem. Eng. Data.

[12] Zhao X., Guo S., Li H., Liu J., Liu X., Song H. (2017). In Situ Synthesis ofImidazolium-CrosslinkedIonogels via Debus-RadziszewskiReactionBasedon PAMAM Dendrimers inImidazoliumIonicliquid. Macromol. Rapid. Commun..

[13] Jaitely V., Karatas A., Florence A.T. (2008). Water immiscible room temperature ionic liquids: Some properties relevant to their pharmaceutical use. Int. J. Pharm..

[14] Mizuuchi H., Jaitely V., Murdan S., Florence A.T. (2008). Room temperature ionic liquids and their mixtures: Potential pharmaceutical solvents. Eur. J. Pharm. Sci..

[15] Badone D., Lau C.K., Jiandong Y. (2014). Aliskirenhemifumarate, Crystal form and Amorphous Solid. US Patent.

[16] Aliskiren (hemifumarate), Cayman Chemicals. https://www.caymanchem.com/product/19640.

[17] Marciniak A. (2011). The Hildebrand Solubility Parameters of Ionic Liquids-Part 2. Int. J. Mol. Sci..

[18] Ruidiaz M.A., Delgado D.R., Martínez F., Marcus Y. (2010). Solubility andpreferential solvation of indomethacin in 1,4-dioxane + water solvent mixtures. Fluid Phase Equailib..

[19] Manrique Y.J., Pacheco D.P., Martínez F. (2008). Thermodynamics of mixing and solvation of ibuprofen and naproxen in propylene glycol + water cosolvent mixtures. J. Sol. Chem..

[20] Shakeel F., Imran M., Haq N.A., Alanazi F.K., Alsarra I.A. (2017). Solubility and thermodynamic/solvation behavior of 6-phenyl-4,5-dihydropyridazin-3(2H)-one in different (Transcutol + water) mixtures. J. Mol. Liq..

[21] Apelblat A., Manzurola E. (1999). Solubilities of o-acetylsalicylic, 4-aminosalicylic, 3,5-dinitrosalicylic and p-toluic acid and magnesium-DL-aspartatein water from T = (278–348) K. J. Chem. Thermodyn..

[22] Manzurola E., Apelblat A. (2002). Solubilities of L-glutamic acid, 3-nitrobenzoicacid, acetylsalicylic, p-toluic acid, calcium-L-lactate, calcium gluconate, magnesium-DL-aspartate, and magnesium-L-lactate in water. J. Chem. Thermodyn..

[23] Holguín A.R., Rodríguez G.A., Cristancho D.M., Delgado D.R., Martínez F. (2012). Solution thermodynamics of indomethacin in propylene glycol + watermixtures. Fluid Phase Equalib..

[24] Krug R.R., Hunter W.G., Grieger R.A. (1976). Enthalpy-entropy compensation. 2. Separation of the chemical from the statistic effect. J. Phys. Chem..

[25] Shakeel F., Haq N., Raish M., Anwer M.K., Al-Shdefat R. (2016). Solubility and thermodynamic analysis of sinapic acid in various neat solvents at different temperatures. J. Mol. Liq..

[26] Wrasse-Sangoi M., Secretti L.T., Diefenbach I.F., Rolim C.M.B., Sangoi M.D. (2010). Development and validation of an UV spectrophotometric method for thedetermination of aliskiren in tablets. Quim. Nova.

[27] Anwer M.K., Al-Mansoor M.A., Jamil S., Al-Shdefat R., Ansari M.N., Shakeel F. (2016). Development and evaluation of PLGA polymer based nanoparticles of quercetin. Int. J. BiolMacromol..

[28] Anwer M.K., Mohammad M., Fatima F., Alshahrani S.M., Aldawasari M.F., Alalaiwe A., Al-Shdefat R., Shakeel F. (2019). Solubility, solution thermodynamics and molecular interactions of osimertinib in some pharmaceutically useful solvents. J. Mol. Liq..

[29] Ahmad A., Raish M., Alkharfy K.M., Alsarra I.A., Ahad A., Jan B.L., Shakeel F. (2018). Solubility, solubility parameters and solution thermodynamics of thymoquinone in different mono solvents. J. Mol. Liq..

[30] Anwer M.K., Al-Shdefat R., Jamil S., Alam P., Abdel-Kader M.S., Shakeel F. (2014). Solubility of Bioactive Compound Hesperidin in Six Pure Solvents at (298.15 to333.15) K. J. Chem. Eng. Data.

